# Combination of iTRAQ proteomics and RNA-seq transcriptomics reveals multiple levels of regulation in phytoplasma-infected *Ziziphus jujuba* Mill

**DOI:** 10.1038/hortres.2017.80

**Published:** 2017-12-27

**Authors:** Xia Ye, Huiyu Wang, Peng Chen, Bing Fu, Mengyang Zhang, Jidong Li, Xianbo Zheng, Bin Tan, Jiancan Feng

**Affiliations:** 1 College of Horticulture, Henan Agricultural University, Zhengzhou 450002, China

## Abstract

Jujube witches’ broom (JWB) is caused by infection with a phytoplasma. A multi-omics approach was taken during graft infection of jujube by JWB-infected scion through the analysis of the plant transcriptome, proteome and phytohormone levels. A high number of differentially expressed genes (DEGs) were identified 37 weeks after grafting (WAG), followed by observation of typical symptoms of JWB at 48 WAG. At 37 WAG, the majority of the upregulated DEGs and differentially expressed proteins (DEPs) were related to flavonoid biosynthesis, phenylalanine metabolism and phenylpropanoid biosynthesis. Two of the four upregulated proteins were similar to jasmonate-induced protein-like. Among the downregulated genes, the two most populated GO terms were plant–pathogen interaction and plant hormone signal transduction (mainly for tryptophan metabolism). Moreover, phytoplasma infection resulted in reduced auxin content and increased jasmonate content, indicating that auxin and jasmonic acid have important roles in regulating jujube responses during the first and second stages of phytoplasma infection. At 48 WAG, the two largest groups of upregulated genes were involved in phenylpropanoid biosynthesis and flavonoid biosynthesis. Both genes and proteins involved in carbon metabolism and carbon fixation in photosynthetic organisms were downregulated, indicating that photosynthesis was affected by the third stage of phytoplasma infection.

## Introduction

Jujube (*Ziziphus jujuba* Mill.) is a major fruit crop cultivated in India, Russia, the Middle East, southern Europe and, especially, China.^1^ Among the diseases of jujube trees, jujube witches’ broom (JWB) is currently the most destructive and devastating disease in Asia.^2,3^ JWB is caused by a phytoplasma that can be transmitted by insect vectors and grafting. The typical symptoms of a phytoplasma-infected jujube include excessive stem production from a single point (witches’ broom), yellowing and floral organs turning into leaf-like structures (phyllody).^2^

Phytoplasmas are important agricultural pathogens^4^ and have been found in over 1000 plant species worldwide.^5^ Recently, phytoplasma effector proteins have been shown to target transcription factors, phytohormone receptors and other components of phytohormone signaling in the host plant in order to modulate plant development.^6–11^ For example, overexpression of the phytoplasma effector SAP54 induces indeterminate leaf-like flower development in Arabidopsis plants.^6^ The effector SAP11 from Aster Yellows Witches’ Broom (AY-WB) Phytoplasma alters Arabidopsis morphology, destabilizes Arabidopsis CINCINNATA (CIN)-related TEOSINTE BRANCHED1, CYCLOIDEA and the PROLIFERATING CELL FACTORS 1 and 2 (TCP) transcription factors, and reduces lipoxygenase (LOX2) gene expression and jasmonate (JA) synthesis.^7^ The TENGU effector causes Arabidopsis sterility by downregulating the JA and auxin pathways.^10^ Together, these results indicate that phytoplasma effectors have vital roles in phytoplasma pathogenesis and host–pathogen interaction. However, phytoplasma effectors have not been identified in woody plants, and it was unknown whether the phytoplasmas that infect trees carry effectors and whether these effectors have similar functions as those in Arabidopsis.

The plant response to phytoplasma infection has also been studied at the physiological and biochemical levels. These studies have shown that phytoplasma infection affects photosynthetic activity,^12^ increases antioxidant enzyme levels and reduces the contents of chlorophyll, total soluble sugars and auxin in infected plants.^13,14^ An imbalance in phytohormones has been suggested to be a main reason for development of phytoplasma-associated symptoms, such as stunting, proliferation and witches’ broom.^14^ Previous studies indicated that biosynthetic pathways for such secondary metabolites as terpenoid indole alkaloids and phenylpropanoids were stimulated by phytoplasma infection.^15^ A few studies have focused on the molecular mechanisms of plant reactions. Using suppressive subtraction hybridization, defense genes such as *Peroxidase*, *Thaumatin-like* protein, *PR10* and *Proline-rich* protein, and *eEF1A* protein were predicated to have an important function in a resistant jujube cultivar in response to phytoplasma infection.^3,16^

On the basis of previous reports, it is clear that the responses of host plants to phytoplasma infection are complex. However, the physiological and molecular mechanisms during disease symptom development are still poorly understood. In this study, we analyzed changes in the transcriptome, proteome and phytohormone levels in response to grafting a phytoplasma-infected scion onto a susceptible cultivar of jujube. The large-scale, multi-omics data set allowed identification of jujube genes and proteins that respond early to phytoplasma infection. Further, we explored the correlation between the phytoplasma-responsive transcriptome and proteome, which can serve as the foundation for further phytoplasma pathogenesis and response mechanism studies.

## Materials and methods

### Plant materials

Two-year-old jujube (*Ziziphus jujuba* Mill. ‘Huizao’) plants were grown in pots in an insect-free net-house at Henan Agricultural University, Zhengzhou, China. Leaves of each jujube plants were collected at three times during spring-summer. DNA was extracted from leaf samples using CTAB^17^ for direct and nested polymerase chain reaction (PCR) using the universal phytoplasma-specific primer sets P1/P7 (ref. 18) and R16F2n/R16R2 (ref. 19) to diagnose phytoplasma infection. Jujube plants that were negative for phytoplasma infection after all three rounds of PCR examination were selected as healthy material for grafting experiments. Buds from JWB-infected adult plants (*Ziziphus jujuba* Mill. ‘Huizao’) were sampled and grafted onto healthy 2-year-old jujube plants on 13 August 2014 in the net-house. In order to analyze the migration of JWB phytoplasma within the host jujube plant, leaves were sampled every 3 days in the first month after grafting and then each week from the second month after grafting (WAG) until JWB symptoms were seen in grafted jujube plants in October 2015.

### PCR analysis

PCR products from the diagnosis PCR using P1/P7 (ref. 18) and R16F2n/R16R2 (ref. 19) primers were sequenced. Primers specific to the 16S rRNA of JWB phytoplasma were designed based on the sequencing results (F1:CGCTAAAGTCCCCACCATTA and R1:CACATTGGGACTGAGACACG). PCR reactions contained 1 μl primer mix (0.5 μM F1 and R1 JWB-specific primers), 100 ng template DNA, 10 μL PCR Master Mix (TaKaRa, Dalian, China) and purified H_2_O water in a total volume of 25 μL. Reactions were conducted using the following thermal cycling conditions: 94 °C for 6 min, followed by 35 cycles of denaturation for 45 s at 94 °C, annealing for 45 s at 56 °C, extension for 1 min at 72 °C and a final extension at 72 °C for 10 min. PCR products (827 bp) were detected using 1.5% agarose gel electrophoresis.

### Sample collection and transcriptome analysis

**Figure 1:**
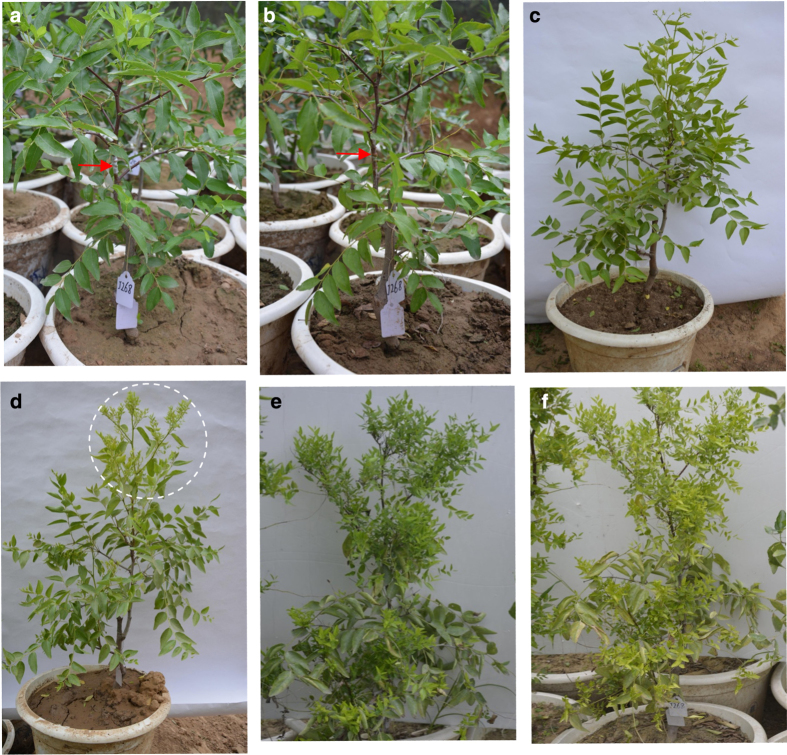
Symptoms of jujube infected by grafted buds carrying JWB phytoplasma at different stages. (**a**) Zero weeks after grafting (WAG); (**b**) 2 WAG; (**c**) 37 WAG; (**d**) 39 WAG; (**e**) 48 WAG; (**f**) 52 WAG. Red arrow: grafting position. The dotted circle: symptoms began to appear.

According to PCR results and symptom observation of grafted plants (Supplementary Figure S1 and Figure 1), leaf samples at six stages after grafting and from healthy plants at the same stages were collected for RNA extraction and transcriptome analysis. The six stages of infection were set at 0 WAG (13 August 2014), 2 WAG (23 August 2014), 37 WAG (27 May 2015), 39 WAG (10 June 2015), 48 WAG (13 August 2015) and 52 WAG (16 September 2015). Three trees in one replicate and three biological replicates were set, and in total nine healthy and nine infected trees were sampled in each treatment. Three or four leaves from each plant were individually collected at each stage, frozen in liquid nitrogen and then stored in −80 °C.

For RNA extraction and transcriptome analysis, nine healthy and nine grafted plants with similarly developing characteristics were selected from more than 100 healthy and JWB-infected jujube plants for this experiment. Three samples were prepared for the following experiments. Total RNA was extracted from the above infected and healthy leaves using the RNAprep Pure Plant Kit (DP441, TianGen, Beijing, China). RNA quality and quantity were determined in a NanoDrop 2000c spectrophotometer (Thermo Scientific, Waltham, MA, USA), and the RNA integrity was assessed by electrophoresis in 1.0% agarose gel. Total RNA was digested with Dnase I to remove DNA. The purified RNA with an *A*_260_/*A*_280_ ratio of 1.8–2.0 was used for transcriptome sequencing. The libraries were constructed and sequenced in an Illumina Hiseq 2500 platform.

Clean data were obtained by removing the two ends of sequences with low quality (threshold value: 30), removing adapter contamination and removing reads with length less than 60 bp. The resulting reads were aligned to the *Z. jujuba* genome^20^ and the mapped results were then subjected to BLAST against the UniProtKB database (www.uniprot.org). Annotation information was obtained and differentially expression genes (DEGs) were screened based on having a fold change ⩾2 and a *P* value <0.05.

### iTRAQ labeling and MS analysis

For iTRAQ proteomics analysis, protein from the above three individual leaf samples (the same as in section 2.3) collected from healthy and diseased plants at 37 WAG and 48 WAG was extracted using SDT lysis and FASP method,^21^ and the detailed procedures were described as follows.

#### Protein extraction

The samples were ground into powder in liquid nitrogen, and then added to trichloroacetic acid (TCA)/acetone (1:9) solution by five-time volume and mixed by vortex. The mixture was placed at −20 °C for 4 h, and centrifuged at 6000 *g* for 40 min at 4 °C, and then the supernatant was discarded. The precipitation was washed with pre-cooling acetone for three times, and then air-dried. The obtained powder was added to 30 times volume of SDT buffer (4% sodium dodecyl sulfate (SDS), 1 mM dithiothreitol (DDT) and 100 mM Tris-HCl, pH 7.6), mixed and boiled for 5 min. The lysate was sonicated and then boiled for 15 min, followed by centrifugation at 14 000 *g* for 40 min.^22^

After the protein extraction, the protein was filtered with 0.22 μm filters, and the filtrate was quantified with the bicinchonininc acid Protein Assay Kit (Bio-Rad, Hercules, CA, USA). Proteins (20 μg) were mixed with 5× loading buffer boiled for 5 min, and then separated on 10% SDS-PAGE gel (constant current 14 mA, 90 min) to detect protein purity. Protein bands were visualized with Coomassie Blue R-250 staining.

#### Filter-aided sample preparation (FASP Digestion)

Protein solution (30 μl) was taken and DTT was added to a 10 mM final concentration, and boiled for 5 min, and then cooled to room temperature. UA buffer (200 μl; 8 M urea, 150 mM Tris-HCl, pH 8.0) was added and centrifuged at 14 000 *g* for 15 min for two times. UA buffer (100 μl; 100 mM iodoacetamide in UA) was added by vortex at 600 r.p.m. for 1 min. The samples were incubated for 30 min in darkness, and centrifuged at 14 000 *g* for 15 min. Dissolution buffer (100 μl; AB SCIEX, Foster City, CA, USA; DS buffer) was added and centrifuged at 14 000 *g* for 15 min for two times. Proteins for each sample were incorporated into 30 μl SDT buffer. Then, 100 μl iodoacetamide (100 mM indole-3-acetic acid in UA buffer) was added to block reduced cysteine residues. Finally, the protein suspensions were digested with 4 μg Trypsin (Promega, Madison, WI, USA) in 40 μl DS buffer overnight at 37 °C, and the resulting peptides were collected as a filtrate.^23^ The filtrated peptides of each sample were desalted on C18 Cartridges (Empore SPE Cartridges C18, bed I.D. 7 mm, volume 3 mL, Sigma, St Louis, MO, USA), concentrated by vacuum centrifugation and reconstituted in 40 μL of 0.1% (v/v) formic acid. The peptide content was estimated by ultraviolet light spectral density at 280 nm using an extinction coefficient of 1.1 of 0.1% (g L^−1^) solution.

#### iTRAQ labeling and strong cation exchange fractionation

The resulting peptide mixture from each sample was labeled using iTRAQ reagent according to the manufacturer’s instructions (Applied Biosystems, Foster City, CA, USA). The leaf samples collected from healthy plants at 37 WAG and 48 WAG were labeled as respective controls.

The iTRAQ-labeled peptides were fractionated by strong cation exchange (SCX) chromatography using the AKTA Purifier system (GE Healthcare, Uppsala, Sweden) by the following steps: reconstituted and acidified the labeled peptides with buffer A (10 mM KH_2_PO_4_ in 25% of acetonitrile, pH 3.0), and then loaded onto a PolySULFOETHYL 4.6×100 mm column (5 μm, 200 Å, PolyLC Inc, Colombia, MD, USA) and eluted at a flow rate of 1 ml min^−1^ with a gradient of 0–8% buffer B (500 mM KCl, 10 mM KH_2_PO_4_ in 25% of acetonitrile, pH 3.0) for 22 min, followed by 8–52% buffer B during 22–47 min, 52–100% buffer B during 47–50 min, 100% buffer B during 50–58 min and finally buffer B was reset to 0% after 58 min. The elution was monitored by measuring the absorbance at 214 nm, and fractions were collected at every 1 min. The eluted peptides were desalted with C18 Cartridges (Empore SPE Cartridges C18, bed I.D.7 mm, volume 3 mL, Sigma) and concentrated by vacuum centrifugation.

#### Mass spectrometry

Each obtained fraction was injected for nano Liquid chromatography-mass spectrometry analysis. The peptide mixture in buffer A (0.1% formic acid) was loaded onto a reverse phase trap column (Acclaim PepMap100, 100 μm×2 cm, nanoViper C18, Thermo Scientific) connected to the C18-reversed phase analytical column (Easy Column, 10 cm long, 75 μm inner diameter, 3 μm resin, Thermo Scientific). Then, the peptide was separated with a linear gradient of buffer B (84% acetonitrile and 0.1% formic acid) at a flow rate of 300 nl min^−1^ controlled by IntelliFlow technology. The linear gradient was: 0–35% buffer B for 50 min, 35–100% buffer B for 5 min and hold in 100% buffer B for 5 min.

LC-MS/MS analysis was performed on a Q Exactive mass spectrometer (Thermo Scientific). MS data were obtained from the survey scan (300–1800 *m*/*z*) for higher-energy-collisional dissociation fragmentation. Automatic gain control target was set to 3e6 and maximum inject time to 10 ms. Dynamic exclusion duration was 40 s. Survey scans were acquired at a resolution of 70 000 at 200 *m*/*z* and resolution for higher-energy-collisional dissociation spectra was set to 17 500 at 200 *m*/*z*, and isolation width was 2 *m*/*z*. Normalized collision energy was 30 eV and the underfill ratio was defined as 0.1%.

#### Data analysis

MS/MS spectra were searched against the UniProtKB database (www.uniprot.org) using the MASCOT engine (Matrix Science, London, UK; version 2.2) embedded into Proteome Discoverer 1.4. Relative parameters was set as follows: trypsin was chosen as the enzyme, and Carbamidomethyl (C), iTRAQ 4/8 plex (N-term) and iTRAQ 4/8 plex (K) as fixed modifications; Oxidation (M) and iTRAQ 4/8plex (Y) as variable modifications; peptide mass tolerance: ±20 mg/l and fragment mass tolerance: 0.1 Da. To reduce the probability of false peptide identification (false discovery rate (FDR)), only peptides with FDR of 1% at the protein level were counted as the identified protein and each identified protein had at least one unique peptide. For protein quantification, the protein ratios are calculated as the median of only unique peptides of the protein. The thresholds of unique peptide were determined by FDR <0.01, and protein was considered as a differentially expressed protein (DEP) if its fold change was at least 1.2 and its *P* value <0.05 (Student’s *t*-test).

### Correlation analysis of transcriptome and proteome

Correlation between the expression levels of a gene in the transcriptome and its corresponding protein in the proteome was evaluated using Spearman’s correlation test.^24^ The results were divided into three categories: the same expression trend, the opposite expression trend and no expression difference.

### Bioinformatics analysis

Annotation analysis of Gene Ontology (GO) was performed for the screened DEGs with the Blast2GO software (http://www.geneontology.org). Following three ontologies for GO annotation of DEGs were included: molecular function, cellular component and biological process. GO enrichment analysis was carried out according to all GO terms that were significantly enriched by the DEGs. For each GO term, the number of genes was calculated before the hypergeometric test to find significantly enriched GO terms based on the input list of DEGs.^25^

KEGG (Kyoto Encyclopedia of Genes and Genomes) databases (http://www.genome.jp/kegg/pathway.html) were used to perform pathway enrichment analysis of the DEGs.^26^

### Phytohormone quantification

The above leaf samples collected at 0 WAG, 37 WAG and 48 WAG were further analyzed for free JA and salicylic acid content using HPLC -MS/MS (high performance liquid chromatography-MS/MS) (SCIEX-6500Qtrap, Applied Biosystems, Foster City, CA, USA) as described.^27^ Phytohormone concentration was analyzed using SPSS 17.0 with three replications, and the Duncan’s multiple range test was applied at *P*=0.05 probability level to evaluate the significant differences among treatments.

### Real-time PCR analysis

Complementary DNA (cDNA) sequences of DEGs were downloaded from transcriptome sequence data, and real-time PCR primers were designed by Primer Express 3.0 (ABI; Supplementary Table S1). Total RNA was reverse-transcribed using PrimeScript RT reagent kit (TaKaRa), and then cDNA solution was diluted to 80–100 μL according to their concentration with Rnase-free water. Real-time PCR was performed with SYBR Premix Ex Taq II kit (TaKaRa) using an ABI PRISM 7500 FAST Sequence Detection System (Applied Biosystems). Reactions of 20 μL total volume contained 1 μL diluted cDNA template, 2 μL primers (0.4 μM each forward and reverse primer), 10 μL SYBR Premix Ex Taq II solution and 7 μL water. The amplification reaction was conducted at 95 °C for 30 s, 40 cycles of 95 °C for 5 s, 60 °C for 31 s and a final dissociation step at 95 °C for 15 s, 60 °C for 1 min and 95 °C for 15 s. Each experiment was repeated three times with three biological replicates.

Relative expression levels of DEGs at 37 WAG infected versus noninfected scions were measured using the ΔΔC_T_ method, and *actin* gene was used as a reference gene for data normalization.

## Results

### Transcriptome and proteome differences during early phytoplasma infection via grafting

PCR analysis (Supplementary Figure S1) was used to determine the infection stages in jujube plants receiving infected bud grafts. The transcriptome of leaf samples from these six stages after grafting indicated that the highest number of DEGs occurred at 37 WAG. Typical symptoms of JWB were observed at 48 WAG (Figure 1). Therefore, leaf samples from the above two stages were sampled for protein analysis using iTRAQ during phytoplasma infection.

Through transcriptome analysis, 25 067 genes were identified at both 37 WAG and 48 WAG (Table 1). Of these, 16 703 and 21 367 genes were annotated in the SwissProt and TrEMBL databases, respectively. At 37 WAG, 1994 genes were significantly differentially expressed in JWB-grafted plants compared with uninfected plants, with 693 of these upregulated and 1301 of these downregulated (fold change >2.0 and *P* values <0.05 in *t*-test (Supplementary Tables S2 and S3)). At 48 WAG, 2401 DEGs were detected, with 808 genes upregulated and 1593 genes downregulated compared with the uninfected controls (Supplementary Tables S4 and S5).

The iTRAQ analysis resulted in a total of 583 908 spectra, with 41 465 of these matching known peptides. Among them, 37 992 unique peptides were identified, and 6748 proteins were explored. At 37 WAG, 5378 proteins were identified, and at 48 WAG 5377 were identified (Table 1). At 37 WAG, a total of 289 differentially expressed proteins (DEPs) were observed, with 176 proteins upregulated and 113 proteins downregulated compared with the uninfected control (fold change >1.2 and FDR <0.01). At 48 WAG, a total of 753 DEPs were detected, with 358 proteins upregulated and 395 proteins downregulated compared with the uninfected control.

### GO analysis of DEGs and DEPs

Of the 25 067 genes identified in the transcriptome analysis, 18 926 genes (75.5%) were annotated via GO analysis. At 37 WAG the category with the most DEGs was cellular components, with 693 genes upregulated with the main functions defined as chloroplast (119 genes) and chloroplast stroma (55 genes). In the molecular function category, most of the upregulated genes were involved in metal ion binding (80 genes) and iron ion binding (29 genes). In the biological process category, 28 upregulated genes were involved in flavonoid biosynthetic process (Supplementary Table S2). Among the 1301 downregulated genes (Supplementary Table S3), 82 genes were involved in response to biotic stimulus, and more than 137 genes were involved in phytohormone regulation (Supplementary Table S3). The top hormone-related functions were auxin-activated signaling pathway (66 genes), response to SA (44 genes) and response to jasmonic acid (27 genes; Supplementary Table S3). Three genes (*XLOC_013752*, *XLOC_013753* and *XLOC_021944*) related to JA O-methyltransferase activity were also downregulated (Supplementary Table S3).

At 48 WAG there were 808 upregulated genes (Supplementary Table S4). The categories with the most DEGs were cellular components, such as the 214 genes encoding integral components of the membrane, 197 genes related to the plasma membrane and 105 genes related to the plasmodesma. Among the 1593 downregulated genes (Supplementary Table S5), the cellular component category was again the most represented, such as the 342 genes involved in chloroplasts, 211 genes related to membranes and 121 genes involved in the chloroplast stroma. Together, this means that the expressions of most genes in the photosynthetic system were affected by phytoplasma infection.

At 37 WAG, 289 DEPs were classified into 43 categories according to their biological process, molecular function or cellular component (Supplementary Figure S2 and Supplementary Table S6). There were 176 proteins upregulated and 113 proteins downregulated compared with the uninfected control (fold change >1.2 and *P* value <0.05 in *t*-test). Among the upregulated DEPs, there were four proteins with abundance changes of more than twofold, namely a JA-induced protein-like (4.38-fold, TCONS_00019449), a granule-bound starch synthase (3.29-fold, TCONS_00052666), a phosphoenolpyruvate carboxykinase-like protein (2.14-fold, TCONS_00033847) and another JA-induced protein-like (2.01-fold, TCONS_00032059; Table 2). Two of the top four DEPs were related to JA-induced protein, in which the co-expression with the JA biosynthesis enzyme (allene oxide synthase) is accompanied by a rise in JAs.^28^ Proteins related to flavonoid biosynthesis (1.89-fold and 1.77-fold higher) and phenylalanine metabolism pathways (1.81-fold) were also upregulated in the leaves of the infected jujube (Table 2). Of the downregulated DEPs, the one with the most change, at more than threefold, was GRF1-interaction factor 1 protein (Table 2), which is a component of the pathway controlling leaf growth by regulating cell proliferation in a transverse direction.

In leaves at 48 WAG, 753 DEPs were detected, with 358 proteins upregulated and 395 proteins downregulated compared with the uninfected control (Supplementary Figure S2 and Supplementary Table S7). Among the upregulated proteins, three of the top 10 DEPs were L-gulonolactone oxidase-like proteins (TCONS_00033106: 3.63-fold, TCONS_00033103: 2.98-fold and TCONS_00033097: 2.73-fold), which are enzymes that produce vitamin C (Table 3). Two of the top 10 upregulated proteins were JA-induced protein-like (TCONS_00032059: 3.16-fold and TCONS_00006683: 2.78-fold). Of the downregulated proteins, a more than sixfold decrease of the linoleate 13S-lipoxygenase 2-like protein (LOX2) was observed in the leaf sample at 48 WAG compared with the control, and two of the top five DEPs were JA-induced protein-like proteins (TCONS_00050299: 0.344 and TCONS_00019449: 0.370), which means JA was an important factor during phytoplasma infection of jujube (Table 3).

### KEGG pathway analysis for DEGs and DEPs

Of the 25 067 genes from the transcriptome analysis, 10 104 genes (40.3%) were annotated by KEGG analysis. At 37 WAG in plants grafted with JWB-infected scions, 20 of the upregulated genes were involved in flavonoid biosynthesis, 11 in phenylpropanoid biosynthesis and 10 genes in phenylpropanoid metabolism (Supplementary Table S8). Among the downregulated genes, the two most populated groups were related to plant–pathogen interaction and plant hormone signal transduction (Supplementary Table S9 and Supplementary Figures S3 and S4). Three to four downregulated genes were involved in plant–pathogen interactions (Table 4 and Supplementary Figure S4), with five of these genes representing two calcium-binding proteins (CML), one LRR receptor-like serine/threonine-protein kinase (FLS2), pathogenesis-related protein 1 (PR1) and the disease resistance protein RPM1 expressed at levels eightfold lower than in the control. A total of 28 genes involved in plant hormone signal transduction were downregulated (Table 5 and Supplementary Figure S-3), with 11 of these genes related to tryptophan metabolism.

At 48 WAG in plants grafted with JWB-infected scions, among the upregulated genes, 16 of the upregulated genes were related to phenylpropanoid biosynthesis and 13 to flavonoid biosynthesis (Supplementary Table S10). Among the downregulated genes at 48 WAG, 74 genes were related to ribosome, 34 genes to carbon metabolism and 24 genes to photosynthesis (Supplementary Table S11).

**Figure 2:**
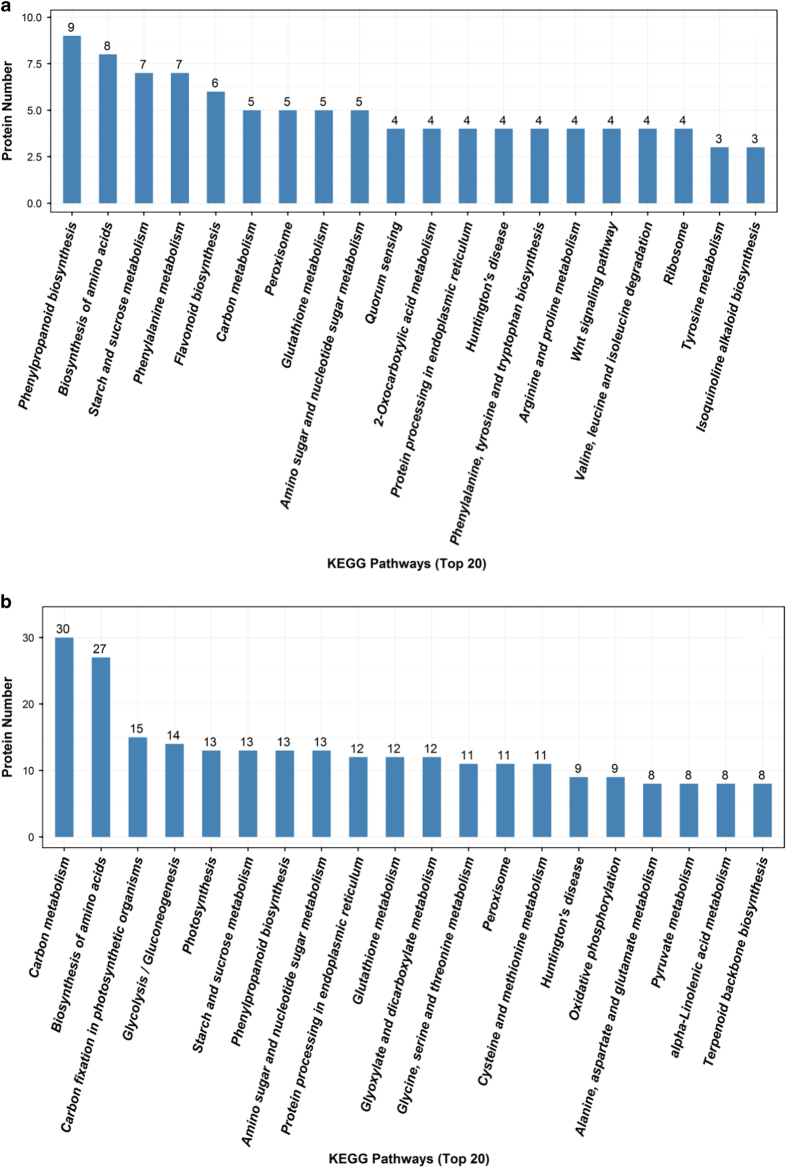
The top 20 enriched KEGG pathways based on DEPs in leaves of jujube during JWB phytoplasma infection. (**a**) 37 WAG infected versus noninfected scions; (**b**) 48 WAG infected versus noninfected scions.

To further investigate the plant reaction to JWB infection, potential biological functions of 289 DEPs from the 37 WAG sample and 753 DEPs from 48 WAG were identified by searching the sequences against the KEGG database. The 289 DEPs from 37 WAG were assigned to 131 KEGG pathways, and the top five pathways with the highest richFactor (numbers of enriched DEPs/annotated proteins in pathway) were phenylpropanoid biosynthesis, biosynthesis of amino acids, starch and sucrose metabolism, phenylalanine metabolism and flavonoid biosynthesis (Figure 2a). The 753 DEPs from 48 WAG were assigned to 195 KEGG pathways, and the DEPs with the highest richFactor were those involved in carbon metabolism, biosynthesis of amino acids and carbon fixation in photosynthetic organisms (Figure 2b).

### Correlation analysis between transcriptome and proteome data

**Figure 3:**
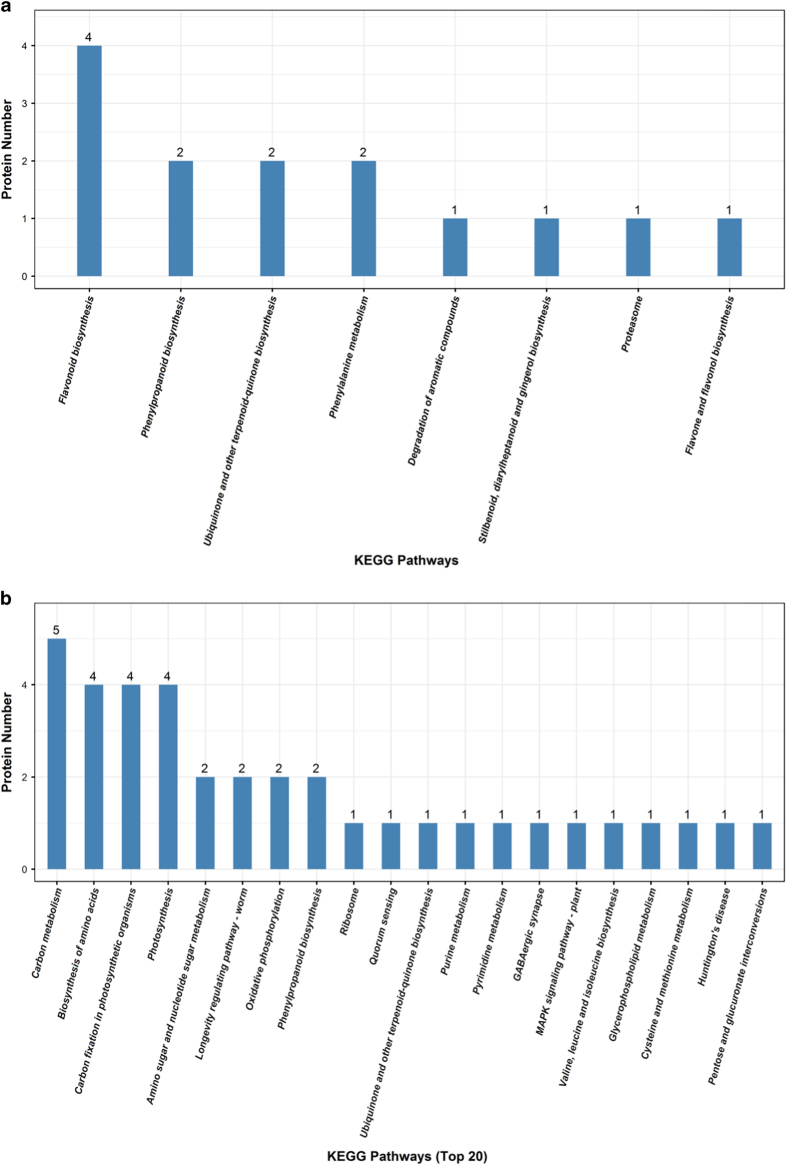
KEGG analyses of DEPs and DEGs with the same expression trend during phytoplasma infection in *Ziziphus jujuba* Mill. ‘Huizao’. (**a**) 37 WAG; (**b**) 48 WAG.

In the leaf samples at 37 WAG, 1367 genes/proteins were correlated in the detected 5378 proteins and 9753 transcripts (with relative expression value >0 in both healthy leaf and diseased leaf samples; Table 6). Among the DEGs/DEPs in the leaf samples at 37 WAG, 70 DEPs showed no corresponding genes in the transcript data and 299 DEGs showed no corresponding proteins in the proteome data (Table 6). Fourteen DEGs/DEPs showed similar expression trends, and most of these were involved in flavonoid biosynthesis, phenylalanine metabolism and phenylpropanoid biosynthesis (Figure 3a).

Of the detected 5377 proteins and 9744 transcripts from leaf samples at 48 WAG, the changes in 1377 genes/proteins were found to be correlated (Table 6). There were 210 DEPs with no corresponding genes in the transcript data, and 377 DEGs with no corresponding proteins in the proteome data (Table 6). Most of 98 DEGs/DEPs with the similar expression trend in the correlation analyses were involved in carbon metabolism, carbon fixation in photosynthetic organisms and photosynthesis (Figure 3b).

### Auxin and JA content analysis

**Figure 4:**
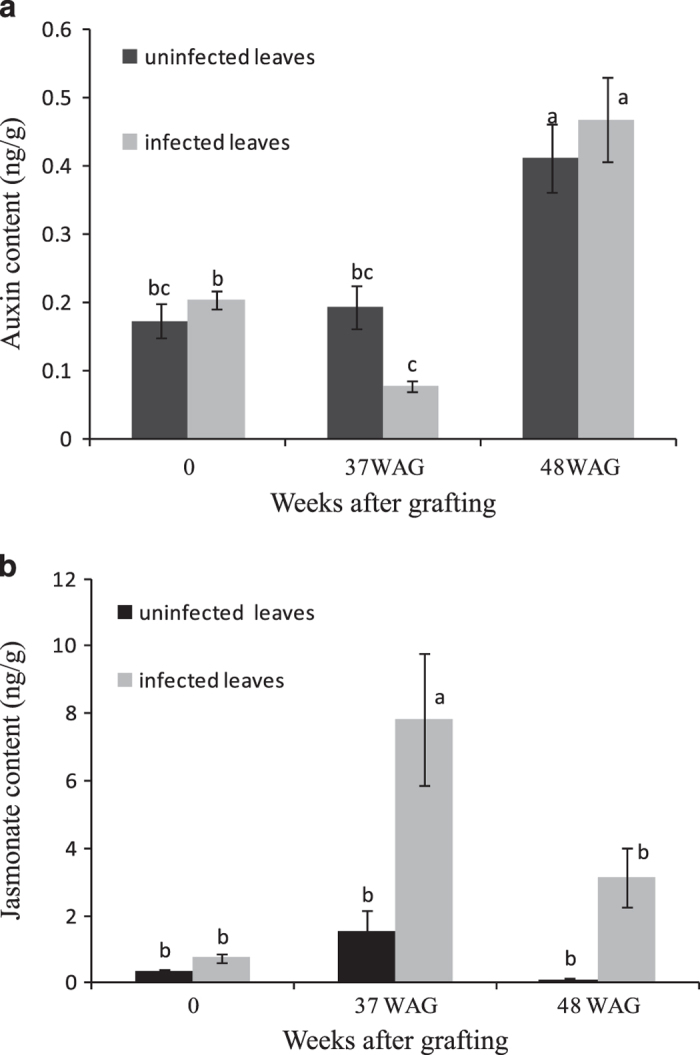
Auxin and jasmonate content in leaf samples 37 and 48 WAG with JWB phytoplasma-infected scions. (**a**) Auxin content; (**b**) Jasmonate content. Letters at same time point indicate differences between infected and uninfected jujube leaves.

According to the transcriptome and proteome analysis, 11 DEGs were related to tryptophan metabolism and the two most-changed DEPs were related to JA-induced protein-like in the leaf samples at 37 WAG. Therefore, the auxin and JA contents were analyzed in the control and diseased leaf samples. The results indicated that phytoplasma infection resulted in reduced auxin content and increased JA content at the early stage of JWB disease (Figure 4). Auxin content in the diseased leaf samples decreased by 2.5-fold compared with the control sample; however, there was no difference in auxin content at 48 WAG between the infected jujube and the corresponding control (Figure 4a). The JA content was higher in the infected jujube at both 37 and 48 WAG, although the amplitudes were different. The JA content in the leaf samples at 37 WAG was more than five times than that in the control; however, there was not a significant difference in JA content between the infected and control samples at 48 WAG (Figure 4b).

### Confirmation of qRT-PCR

**Figure 5:**
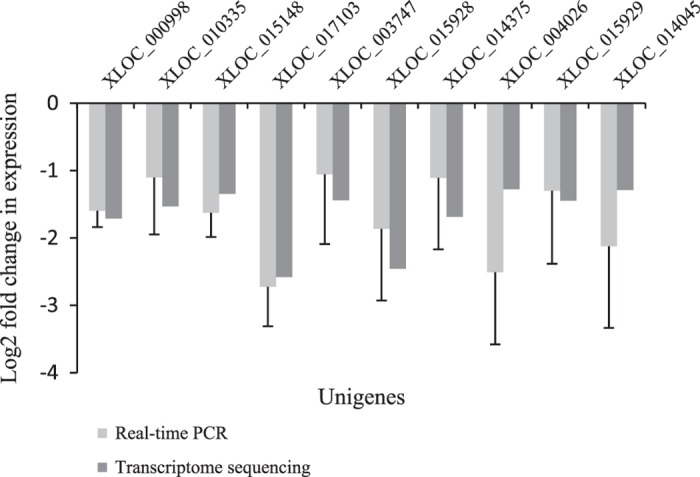
Real-time PCR validation of the relative expression levels of 10 DEGs in the diseased and control leaves at 37 WAG. Expression profiles of selected genes as determined by real-time PCR (grey) and transcriptome sequencing (dark grey). Relative expression of each transcript was normalized using *Actin* gene. The *y* axis shows the normalized expression level of the transcript. The *x* axis indicates genes. Error bars represent the s.d.’s of real-time PCR signals.

In order to evaluate our transcriptome-sequencing data, 10 DEGs in the tryptophan metabolism pathway were selected for quantitative reverse transcriptase PCR (qRT-PCR). The results of qRT-PCR and the transcriptome-sequencing analyses indicated that all tested 10 DEGs showed similar trends in the relative expression levels (Figure 5). For instance, log2 fold change of relative expression levels from XLOC_000998 gene was close to −1.7 in both qRT-PCR and transcriptome-sequencing data, which suggested that the gene expression changes detected by transcriptome-sequencing analysis were reliable.

## Discussion

### Genes or proteins involved in phenylpropanoid biosynthesis or metabolism were upregulated in the early stages after JWB phytoplasma infection

Phenylpropanoid compounds are induced in response to microbial attack and can inhibit the growth of attacking pathogens.^29^ Phenylpropanoids include lignins, flavonoids and phenolic compounds that are products of multiple branches of the phenylpropanoid pathway.^29,30^ In the transcriptome of paulownia infected with witches’ broom phytoplasma, numerous phenylpropanoid metabolism genes were upregulated, including phenylalanine ammonia lyase (PAL), cinnamate 4-hydroxylase and 4-coumarate-CoA ligase.^30^ In our study, 22 genes involved in phenylpropanoid biosynthesis or metabolism were upregulated in the diseased leaf sample at 37 WAG compared with the uninfected control. For instance, expression levels of PAL (XLOC_022321) cinnamoyl-CoA reductase (XLOC_001983) and caffeoyl shikimate esterase (XLOC_003432) were significantly higher than those in the control (Supplementary Tables S8 and S12). This differential expression correlated to the change at 37 WAG in the protein level of PAL (TCONS_00048113) in infected leaves compared with the control (Table 4), which further verifies that phenylpropanoid compounds have important roles in the defense against phytoplasma infection.

Flavonoids are a diverse group of phenolic secondary metabolites and have important roles in plant defenses against both abiotic and biotic stresses.^29^ Flavonoids can function as passive or inducible barriers, and both flavonoid content and expression of genes in the flavonoid biosynthesis pathway increase in response to pathogen attack.^31,32^ In previous transcriptome analyses of phytoplasma infection, flavonoid metabolism-related genes are activated in phytoplasma-infected plants.^30,33^ In our study, more than 20 genes involved in flavonoid biosynthesis were upregulated in the diseased leaf sample at 37 WAG compared with the control. For instance, the relative expression of flavonoid 3'-monooxygenase (XLOC_008694) and anthocyanidin reductase (XLOC_000417) were roughly eight times higher than those in the control (Supplementary Table S8 and Supplementary Table S12). The enzymes dihydroflavonol 4-reductase (TCONS_00024299) and flavonoid 3-hydroxylase (TCONS_00021256), both involved in flavonoid biosynthesis, were also upregulated (Table 2), which further supports that flavonoid biosynthesis has a role in early defense responses against biotic stress.^32^

### Genes involved in tryptophan metabolism and auxin production and signaling were downregulated in the early stages after JWB phytoplasma infection

Auxin imbalance has been suggested as a key factor in the development of symptoms during phytoplasma infection.^14^ For example, levels of indole-3-acetic acid are reduced in mulberry infected with mulberry dwarf phytoplasma.^34^ Furthermore, spraying of 1-naphthaleneacetic acid on periwinkle infected with phytoplasma accelerated symptom development, which led to the conclusion that auxin may suppress expression of genes such as *PATHOGENESIS-RELATED 1* in phytoplasma-infected plants and thus allow more rapid symptom development.^14^ In contrast, when *in vitro*-grown periwinkle shoots infected with ‘*Candidatus* Phytoplasma’ were treated with IAA and indole-3-butyric acid, both auxins induced recovery of phytoplasma-infected periwinkle, which implies that auxin may delay some species of phytoplasma.^35^ This result was further verified when pretreatment of 1-naphthaleneacetic acid also effectively enhanced plant resistance against phytoplasma.^14^ In addition, the phytoplasma virulence factor TENGU was found to repress auxin response factor 6 (ARF6) and ARF8, which then resulted in downregulation of auxin levels.^10^

In this study, 12 genes related to auxin signaling were downregulated at 37 WAG (Table 5), and auxin content declined by 2.5-fold in the phytoplasma-infected leaf samples compared with the control (Figure 4a). At 48 WAG, when typical JWB phytoplasma symptoms were visible, there were no significant differences in the relative expression of genes related to auxin signaling or in auxin content. These results implied that auxin imbalance was a key factor in the early, but not later, stages of JWB phytoplasma infection.

### Levels of JA-induced proteins and JA were altered during JWB phytoplasma infection

The activation of phytohormone signaling pathways is a universal defense response employed by plants in response to biotic or abiotic stress,^36^ and JA has central roles in regulating plant responses to herbivorous insects and microbial pathogens.^37,38^ For example, when *Nicotiana attenuata* is attacked by *M. sexta* larvae, there is a sustained JA burst ninefold above control levels.^36^ *Fusarium oxysporum* pathogens appear to utilize JAs as effectors, promoting both infection in roots and development of symptoms in shoots.^39^ In the Arabidopsis plants infected by AY-WB phytoplasma, the secreted AY-WB protein 11 (SAP11) is an effector that interferes with JA biosynthesis by binding and destabilizing class II CIN-TCP (CINCINNATA-RELATED-TEOSINTE BRANCHED1, CYCLOIDEA, PCF) transcription factors, which normally act as positive regulators of the *LOX2* gene involved in JA biosynthesis.^40^ Moreover, the SAP54 effector produced by AY-WB phytoplasma ^6^ and the TENGU effector produced by onion yellows phytoplasma ^10^ were also found to regulate both JA biosynthesis and levels in phytoplasma-infected plants. All the above research indicates that JA regulation has a vital part in response to herbivorous insects and microbial pathogens.

In our results at 37 WAG, two proteins (TCONS_00019449 and TCONS_00032059, Table 2) involved in α-linolenic acid metabolism as well as JA levels were significantly induced compared with the corresponding levels in the control (Table 2, Figure 4). In our experimental design, 37 WAG occurred in the spring following infection by JWB phytoplasma as new leaves were emerging. At this point, typical disease symptoms were not yet observed in the jujube plants, which implied that upregulation of JA-induced proteins and JA levels had an important part in defending against JWB phytoplasma infection at the early stage.

However, JA levels began to decrease after 37 WAG and consistently declined until 52 WAG (data not shown). There was no significant difference in JA accumulation at 48 WAG between the infected and uninfected jujube plants, when the typical JWB phytoplasma symptoms occurred (Figures 1 and 4). Furthermore, the expression level of linoleate 13S-lipoxygenase 2-like protein, which encodes a key JA biosynthesis enzyme, was six times lower than in the control, and two JA-induced proteins (TCONS_00050299 and TCONS_00019449, which were upregulated at 37 WAG) were downregulated at 48 WAG. These results further verified the tendency that JA biosynthesis and JA levels are disrupted during phytoplasma infections, and that this disruption may be beneficial for increased colonization by the phytoplasmas.^10^

### A hypothetical working process after JWB phytoplasma infection

Two models of innate immunity have been reported in plants.^41,42^ In one model, resistance is triggered by microbe-associated molecular patterns and is referred to as pattern-triggered immunity. In the second model, dubbed effector-triggered immunity, the plant response is triggered by pathogen effectors.^40^ In plants, one of the best-studied pattern-triggered immunity systems is perception of the bacterial flagellin or the flg22 peptide by FLAGELLIN-SENSING 2 (FLS2).^43–45^ Upon perception, the receptor kinase FLS2 mediates initiation of flg22-signaling responses, which contributes to bacterial growth restriction.^43–45^

**Figure 6:**
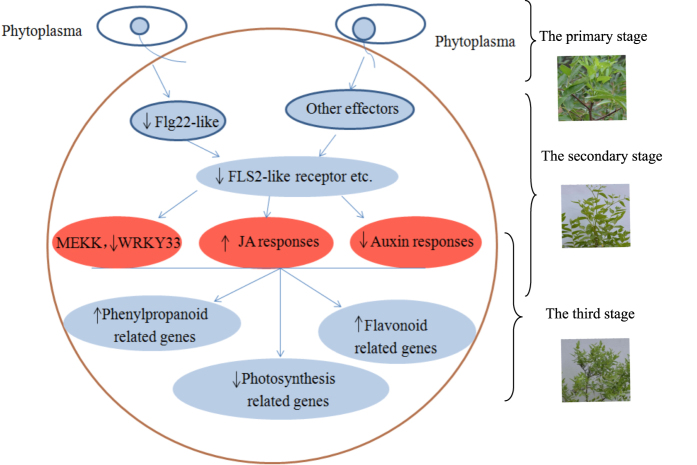
A hypothetical working process after JWB phytoplasma infection. During the primary stage, the phytoplasma is invading the plant and early perception has been activated. Phytoplasma gene could be detected by PCR. By the secondary stage the plant has mounted its defense response. JA-related proteins and JA levels are increasing, whereas auxin-related genes and levels are decreasing. There is a general downregulation of genes involved in plant–pathogen interaction and upregulation of genes involved in phenylpropanoid biosynthesis or metabolism. In the third stage, symptoms appear in the sensitive phenotype, concomitant with decreases in JA-related proteins and JA levels and photosynthesis-related genes. ↑ represented upregulation, ↓ represented downregulation.

Although several effectors, such as SAP11, SAP54 and TENGU, were verified in plants infected by phytoplasmas,^6,10,40^ we have not yet identified any of these above effectors in jujube plants infected with JWB phytoplasma by homologous cloning. However, four receptor kinase FLS2 genes were downregulated at 37 WAG (Table 4), which implied that FLS2/flg22 perception within the pattern-triggered immunity system may exist in the jujube–phytoplasma interaction. From this, we developed a hypothetical working process in which JWB phytoplasma first produces a flg22-like effector after invading jujube plants (the primary stage, Figure 6) that can be perceived by FLS2. However, by 37 WAG, expression levels of the FLS2-like receptors were downregulated in order to mediate perception of flg22-like effector to inhibit colonization of jujube with JWB phytoplasmas (the secondary stage: defense stage, Figure 6). Moreover, JA-related proteins and JA content in leaf samples were increasing, auxin-related genes and content were decreasing and DEGs involved in plant–pathogen interaction and phenylpropanoid biosynthesis or metabolism were downregulated and upregulated, respectively, in the secondary stage. After pathogen recognition, the mitogen-activated protein kinases were activated, followed by regulated expression of transcription factors such as WRKY33. Other related defense genes were further regulated in the third stage (Supplementary Figure S4, Table 4 and Figure 6). In addition, typical JWB symptoms were observed in the sensitive jujube genotype when JA-related proteins and content were decreasing and DEGs involved in photosynthesis were downregulated, which would allow colonization of the jujube plants with JWB phytoplasma.

## Conclusions

To understand the responses and defenses of jujube plants to phytoplasma infection, a multi-omics analysis of transcriptome, proteome and phytohormone levels was conducted during JWB infection. Our results indicated that an increase in the number of DEGs occurred by 37 WAG, but that JWB typical symptoms were not observed until 48 WAG.

At 37 WAG, 1994 genes were significantly differentially expressed. Among the upregulated genes at 37 WAG, 20 genes were assigned to flavonoid biosynthesis and 21 to phenylpropanoid biosynthesis and metabolism. Among the downregulated genes, the top two groups were related to plant–pathogen interaction and plant hormone signal transduction. At 37 WAG, 289 DEPs were observed. Among the upregulated DEPs at 37 WAG were proteins related to JA-induced protein, flavonoid biosynthesis and phenylalanine metabolism. Fourteen DEGs/DEPs were shown to share similar expression trends by correlation analyses, and most of these were involved in flavonoid biosynthesis, phenylalanine metabolism and phenylpropanoid biosynthesis. Moreover, phytoplasma infection resulted in reduced auxin content and increased JA content during the early stage of phytoplasma infection.

At 48 WAG, 2401 DEGs were detected. Among the 808 upregulated genes, many were involved in phenylpropanoid biosynthesis and flavonoid biosynthesis. Among the 1593 downregulated genes, most were related to photosynthesis and carbon metabolism. At 48 WAG, 753 DEPs were detected. Of the 395 downregulated proteins, LOX2 were decreased by sixfold, and two JA-induced proteins were greatly reduced. Moreover, DEPs involved in carbon metabolism, biosynthesis of amino acids and carbon fixation in photosynthetic organisms were also downregulated. At 48 WAG, most of 98 DEGs/DEPs of similar expression trends in the correlation analyses were involved in carbon metabolism, carbon fixation in photosynthetic organisms and photosynthesis.

## Figures and Tables

**Table 1:** Summary of the number of proteins and mRNA detected during phytoplasma infection of *Ziziphus jujuba* Mill.

Category	*Proteins*		*mRNAs*	
	*37 WAG*	*48 WAG*	*37 WAG*	*48 WAG*
Unique protein/gene detected	5378	5377	25 067	25 067
				
*Significantly changed proteins/genes*	289	753	1994	2401
Upregulated	176	358	693	808
Downregulated	113	395	1301	1593

Abbreviation: WAG, weeks after grafting.

**Table 2:** Summary of the top 10 DEPs at 37 WAG with phytoplasma infecting scion

*Accession*^a^	*Sequence description*	*Fold change (37 WAG/CK)*	*KEGG term (Map name)*	P *value*
TCONS_00019449	23 kDa jasmonate-induced –like	4.38	α-Linolenic acid metabolism	0.0419
TCONS_00052666	Granule-bound starch synthase	3.29	Starch and sucrose metabolism	0.0014
TCONS_00033847	Phosphoenolpyruvate carboxykinase [ATP]-like	2.14	None	0.0102
TCONS_00032059	23 kDa jasmonate-induced –like	2.014	α-Linolenic acid metabolism	0.0083
TCONS_00024299	Dihydroflavonol 4-reductase	1.89	Flavonoid biosynthesis	0.0152
TCONS_00048113	Phenylalanine ammonia lyase	1.819	Phenylalanine metabolism	0.0124
TCONS_00021256	Flavonoid 3-hydroxylase	1.77	Flavonoid biosynthesis	0.0128
TCONS_00025885	Aspartic proteinase nepenthesin-2	1.77	None	0.0049
TCONS_00014361	Superoxide dismutase [Fe] chloroplastic-like	1.77	MAPK signaling pathway	0.0094
TCONS_00030498	Desacetoxyvindoline 4	1.75	None	0.0012
TCONS_00008208	GRF1 interaction factor 1	0.31	None	0.0020
TCONS_00000288	DNA replication licensing factor MCM6-like	0.45	DNA replication	0.0395
TCONS_00025058	Nitrate reductase	0.52	Nitrogen metabolism	0.0257
TCONS_00045226	Abscisic acid receptor PYR1-like	0.60	MAPK signaling pathway	0.0019
TCONS_00030214	Sieve element occlusion a	0.61	None	0.0302
TCONS_00034614	Subtilisin-like protease-like	0.61	None	0.0001
TCONS_00022334	Cytochrome P450 71A1-like	0.62	None	0.0054
TCONS_00022924	Movement-binding isoform 1	0.631	None	0.0163
TCONS_00000768	BRG-1 associated	0.65	None	0.0485
TCONS_00009541	125 kDa kinesin-related-like	0.65	None	0.0039

Abbreviations: DEP, differentially expressed protein; KEGG, Kyoto Encyclopedia of Genes and Genomes; MAPK, mitogen-activated protein kinase; WAG, weeks after grafting; CK, controls.

a Accession numbers in our database was available in Supplementary Table S13.

**Table 3:** Summary of the top 10 DEPs at 48 WAG with phytoplasma-infected scion

*Accession*^a^	*Sequence description*	*Fold change (37 WAG/CK)*	*KEGG term (Map name)*	*Pathway ID*	P *value*
TCONS_00023218	B-cell receptor-associated 31-like	4.08	None	None	0.0113
TCONS_00033106	L-gulonolactone oxidase-like	3.63	None	None	0.0005
TCONS_00042803	Beta expansin-like	3.26	None	None	0.0062
TCONS_00032059	23 kDa jasmonate-induced-like	3.16	None	None	0.0003
TCONS_00033103	L-gulonolactone oxidase-like	2.98	None	None	0.0022
TCONS_00055463	Adenylate kinase B-like	2.91	Purine metabolism	K00939	0.0072
TCONS_00022833	1,3-beta-D-glucanase GH17_44	2.84	None	None	0.0110
TCONS_00006683	23 kDa jasmonate-induced-like	2.78	None	None	0.0005
TCONS_00033097	L-gulonolactone oxidase-like	2.73	None	None	0.0495
TCONS_00033649	E3 ubiquitin-ligase HERC2-like	2.66	None	None	0.0478
TCONS_00008624	Linoleate 13S-lipoxygenase 2-like	0.16	Linoleic acid metabolism	K00454	0.0009
TCONS_00050299	23 kDa jasmonate-induced-like	0.34	None	None	0.0019
TCONS_00028902	Sieve element occlusion b Zeatin	0.36	None	None	0.0047
TCONS_00015275	O-glucosyltransferase-like	0.36	None	None	0.0001
TCONS_00019449	23 kDa jasmonate-induced-like	0.37	None	None	0.0077
TCONS_00007094	Dehydrodolichyl diphosphate synthase 2-like	0.38	Terpenoid backbone biosynthesis	K11778	0.0153
TCONS_00010120	Tetratricopeptide repeat-like	0.39	None	None	0.0075
TCONS_00022334	Cytochrome P450 71A1-like presequence protease	0.39	None	None	0.0011
TCONS_00009593	Chloroplastic/mitochondrial-like	0.39	None	None	0.0109
TCONS_00028899	Sieve element occlusion a *n* characterized protein	0.42	None	None	0.0016
TCONS_00000655	Ycf23-like	0.42	None	None	0.0088

Abbreviations: DEP, differentially expressed protein; KEGG, Kyoto Encyclopedia of Genes and Genomes; WAG, weeks after grafting.

a Accession numbers in our database are available in Supplementary Table S13.

**Table 4:** Downregulated genes’ plant–pathogen interaction in KEGG pathway analysis in leaves 37 WAG with JWB-infected scions

*Gene_Id*^a^	*Value_1*	*Value_2*	*Log2 (fold-change)*	*Name*	*Definition*	P *value*
XLOC_008034	52.26	1.99	−4.72	*CML*	Calcium-binding protein CML	0.00005
XLOC_002521	5.97	0.45	−3.72	*FLS2*	LRR receptor-like serine/threonine-protein kinase FLS2	0.00005
XLOC_016194	63.57	5.81	−3.45	*CML*	Calcium-binding protein CML	0.00005
XLOC_016016	100.55	10.81	−3.22	*PR1*	Pathogenesis-related protein 1	0.00005
XLOC_020804	2.59	0.30	−3.10	*RPM1, RPS3*	Disease resistance protein RPM1	0.00165
XLOC_016017	36.10	4.53	−2.99	*PR1*	Pathogenesis-related protein 1	0.0001
XLOC_018443	7.80	1.25	−2.64	*CNGF*	Cyclic nucleotide-gated channel, other eukaryote	0.00145
XLOC_012207	26.92	4.42	−2.61	*CPK*	Calcium-dependent protein kinase	0.00005
XLOC_001631	54.77	9.31	−2.56	*EFR*	LRR receptor-like serine/threonine-protein kinase EFR	0.00005
XLOC_003968	7.321	1.35	−2.44	*FLS2*	LRR receptor-like serine/threonine-protein kinase FLS2	0.0007
XLOC_008782	6.29	1.31	−2.27	*RPM1, RPS3*	Disease resistance protein RPM1	0.0047
XLOC_020805	15.46	3.27	−2.25	*RPM1, RPS3*	Disease resistance protein RPM1	0.00005
XLOC_007998	71.01	15.09	−2.24	*CML*	Calcium-binding protein CML	0.00005
XLOC_012794	500.26	117.36	−2.099	*CETN1*	Centrin-1	0.00005
XLOC_021743	18.13	4.396	−2.06	*FLS2*	LRR receptor-like serine/threonine-protein kinase FLS2	0.00005
XLOC_002411	131.83	33.30	−1.99	*CML*	Calcium-binding protein CML	0.0018
XLOC_004660	10.98	2.82	−1.97	*ERF1*	Ethylene-responsive transcription factor 1	0.00295
XLOC_014256	157.32	42.25	−1.90	*CML*	Calcium-binding protein CML	0.00005
XLOC_016219	3.79	1.102	−1.78	*FLS2*	LRR receptor-like Serine/threonine-protein kinase FLS2	0.00255
XLOC_017988	48.81	15.31	−1.68	*CML*	Calcium-binding protein CML	0.00005
Gene_Id	*Value_1*	*Value_2*	*Log2 (fold-change)*	*Name*	*Definition*	P *value*
XLOC_019723	29.87	9.43	−1.67	*CALM*	Calmodulin	0.0003
XLOC_022437	141.01	44.59	−1.67	*SERK1*	Somatic embryogenesis receptor kinase 1	0.00005
XLOC_007823	48.72	15.44	−1.66	*CML*	Calcium-binding protein CML	0.0018
XLOC_020251	14.76	4.83	−1.62	*RPS2*	Disease resistance protein RPS2	0.00005
XLOC_007197	18.94	6.32	−1.58	*EDS1*	Enhanced disease susceptibility 1 protein	0.00005
XLOC_015508	105.62	36.09	−1.55	*SERK1*	Somatic embryogenesis receptor kinase 1	0.00005
XLOC_007196	64.03	22.91	−1.48	*EDS1*	Enhanced disease susceptibility 1 protein	0.00005
XLOC_011916	11.73	4.25	−1.46	*EFR*	LRR receptor-like Serine/threonine-protein kinase EFR	0.00015
XLOC_003222	7.10	2.93	−1.28	*PBS1*	Serine/threonine-protein kinase PBS1	0.0061
XLOC_022668	3.95	1.65	−1.26	*RBOH*	Respiratory burst oxidase	0.00495
XLOC_012474	30.53	14.37	−1.09	*CML*	Calcium-binding protein CML	0.00165
XLOC_018434	23.35	11.01	−1.09	*WRKY33*	WRKY transcription factor 33	0.00125
XLOC_008767	157.13	77.49	−1.02	*EDS1*	Enhanced disease susceptibility 1 protein	0.0013
XLOC_006178	1.95	0	None	*CERK1*	Chitin elicitor receptor kinase 1	0.00005

Abbreviations: DEP, DEP, differentially expressed protein; KEGG, Kyoto Encyclopedia of Genes and Genomes; JWB, Jujube witches' broom; WAG, weeks after grafting.

a Gene Id is available in our database in Supplementary Table S12.

**Table 5:** Downregulated genes assigned to plant hormone signal transduction in KEGG pathway analysis in leaves 37 WAG with phytoplasma-infected scions

*Gene Id*^a^	*Value 1*	*Value 2*	*Log2 (fold-change)*	*Name*	*Definition*	P *value*	*Pathway*
XLOC_000998	12.44	3.80	−1.71	*IAA*	Auxin-responsive protein IAA	0.00285	Tryptophan metabolism
XLOC_010335	10.40	3.60	−1.53	*IAA*	Auxin-responsive protein IAA	0.00515	Tryptophan metabolism
XLOC_004026	35.57	14.69	−1.28	*IAA*	Auxin-responsive protein IAA	0.00005	Tryptophan metabolism
XLOC_015148	1014.82	399.50	−1.348	*IAA*	Auxin-responsive protein IAA	0.0014	Tryptophan metabolism
XLOC_017103	32.11	5.37	−2.58	*ARR-B*	Two-component response regulator ARR-B family	0.00005	Tryptophan metabolism
XLOC_003747	87.22	32.11	−1.45	*ARR-B*	Two-component response regulator ARR-B family	0.00005	Tryptophan metabolism
XLOC_015928	8.72	1.59	−2.46	*SAUR*	SAUR family protein	0.0052	Tryptophan metabolism
XLOC_014375	48.45	15.05	−1.69	*SAUR*	SAUR family protein	0.0001	Tryptophan metabolism
XLOC_015929	19.78	7.26	−1.45	*SAUR*	SAUR family protein	0.00065	Tryptophan metabolism
XLOC_014045	154.94	63.46	−1.29	*SAUR*	SAUR family protein	0.00005	Tryptophan metabolism
XLOC_015923	23.41	9.761	−1.26	*SAUR*	SAUR family protein	0.00565	Tryptophan metabolism
XLOC_024894	12.04	4.81	−1.32	*CRE*	Histidine kinase 2/3/4 (cytokinin receptor)	0.00655	Zeatin biosynthesis
XLOC_004003	53.82	17.52	−1.62	*AHP*	Histidine-containing phosphotransfer protein	0.00345	Zeatin biosynthesis
XLOC_021061	70.86	25.54	−1.47	*AHP*	Histidine-containing phosphotransfer protein	0.00005	Zeatin biosynthesis
XLOC_011848	105.72	47.43	−1.16	*AHP*	Histidine-containing phosphotransfer protein	0.0002	Zeatin biosynthesis
XLOC_018670	35.22	12.97	−1.44	*PYL*	Abscisic acid receptor PYR/PYL family	0.00005	Carotenoid biosynthesis
XLOC_013439	103.36	47.76	−1.11	*PYL*	Abscisic acid receptor PYR/PYL family	0.0003	Carotenoid biosynthesis
XLOC_022737	4.46	0.33	−3.74	*PP2C*	Protein phosphatase 2C	0.00045	Carotenoid biosynthesis
XLOC_024553	22.15	6.78	−1.71	*EIN3*	Ethylene-insensitive protein 3	0.006	Cysteine and methionine metabolism
XLOC_018626	63.29	30.85	−1.04	*ERF2*	Ethylene-responsive transcription factor 2	0.0007	Cysteine and methionine metabolism
XLOC_015742	4.79	0	None	*ERF2*	Ethylene-responsive transcription factor 2	0.0005	Cysteine and methionine metabolism
XLOC_004660	10.98	2.82	−1.96	*ERF1*	Ethylene-responsive transcription factor 1 protein	0.00295	Cysteine and methionine metabolism
XLOC_024701	21.33	4.01	−2.41	*BRI1*	Brassinosteroid insensitive 1 protein	0.00005	Brassinosteroid biosynthesis
XLOC_009027	244.49	74.01	−1.72	*BRI1*	Brassinosteroid insensitive 1 protein	0.00005	Brassinosteroid biosynthesis
XLOC_003968	7.32	1.35	−2.44	*BRI1*	Brassinosteroid insensitive 1	0.0007	Brassinosteroid biosynthesis
XLOC_016016	100.55	10.81	−3.22	*PR1*	Pathogenesis-related protein 1	0.00005	Phenylalanine metabolism
XLOC_016017	36.10	4.53	−2.99	*PR1*	Pathogenesis-related protein 1	0.0001	Phenylalanine metabolism
XLOC_014850	13.00	2.74	−2.24	*CYCD3*	Cyclin D3	0.00115	Brassinosteroid biosynthesis

a Gene Id is available in our database in Supplementary Table S12.

**Table 6:** Correlation of DEGs and DEPs detected during phytoplasma infection of *Z. jujuba* Mill.

*Category*	*37 WAG*	*48 WAG*
Total numbers of detected transcripts^a^	9753	9744
Total numbers of detected protein	5378	5377
Numbers of correlated genes/proteins	1367	1377
Shared DEPs/DEGs with the similar expression trend	14	98
Shared DEPs/DEGs with the opposite expression trend	23	12
DEPs with no corresponding DEGs	70	210
DEGs with no corresponding DEPs	299	377

Abbreviations: DEG, differentially expressed gene; DEP, differentially expressed protein; WAG, weeks after grafting.

a Number of transcripts with relative expression value >0 in both healthy leaf and diseased leaf samples.
