# Supplementary material for: Combination of iTRAQ proteomics and RNA-seq transcriptomics reveals multiple levels of regulation in phytoplasma-infected Ziziphus jujuba Mill
Source: Hortic Res. 2017 Dec 27;4:17080–. doi: 10.1038/hortres.2017.80 (PMC5744194; doi:10.1038/hortres.2017.80)
Supplement: Supplementary Figure S-1 [file hortres201780-s1.docx]

**Figure S-1 PCR analysis of jujube leaf samples infected by JWB phytoplasma in different stages.** A. 0 WAG; B. 2 WAG; C. 37 WAG; D.39 WAG; E. 48WAG; F.52 WAG. M: DL 2000 marker, 1-20: leaf samples, 21: positive samples, 22: negative samples, 23: dd H_2_O.

A


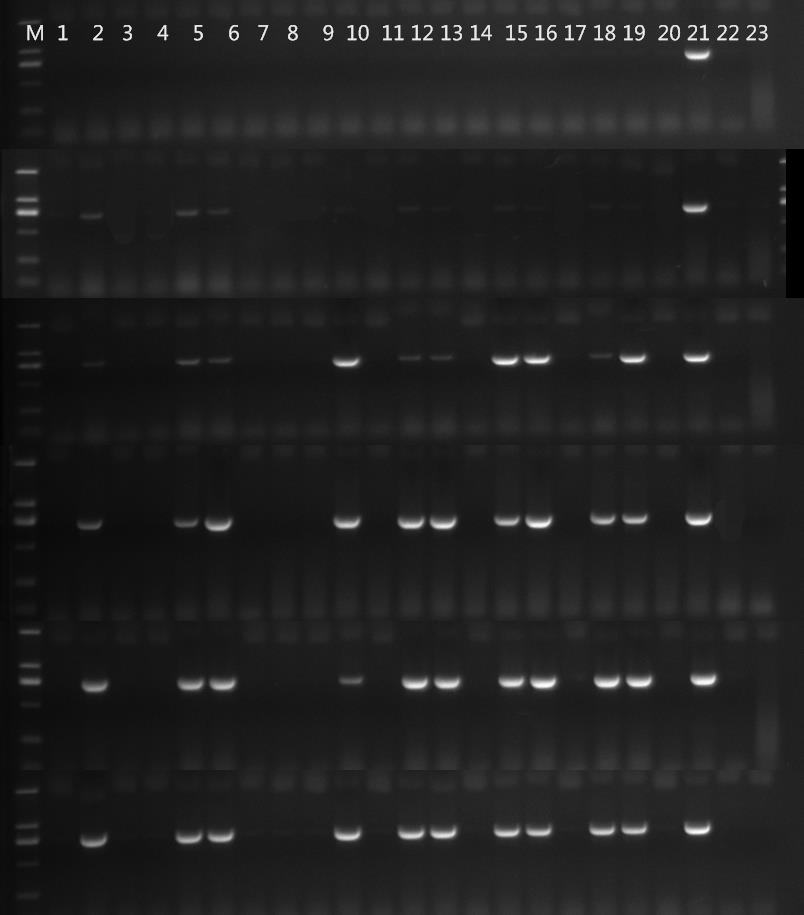


750bp

1000bp

B

C

D

E

F
