# Supplementary material for: Combination of iTRAQ proteomics and RNA-seq transcriptomics reveals multiple levels of regulation in phytoplasma-infected Ziziphus jujuba Mill
Source: Hortic Res. 2017 Dec 27;4:17080–. doi: 10.1038/hortres.2017.80 (PMC5744194; doi:10.1038/hortres.2017.80)
Supplement: Supplementary Figure S-2 [file hortres201780-s2.docx]

**Figure S-2. Gene Ontology (GO) annotation of differentially accumulated proteins (DEPs) in leaves of jujube during JWB phytoplasma infecting.** A. GO annotation of DAPS of 37 WAG infected vs noninfected scions; B. Enriched top 20 GO term of 37 WAG infected vs noninfected scions; C. GO annotation of DAPS of 48 WAG infected vs noninfected scions; D. Enriched top 20 GO term of 48 WAG infected vs noninfected scions.

**
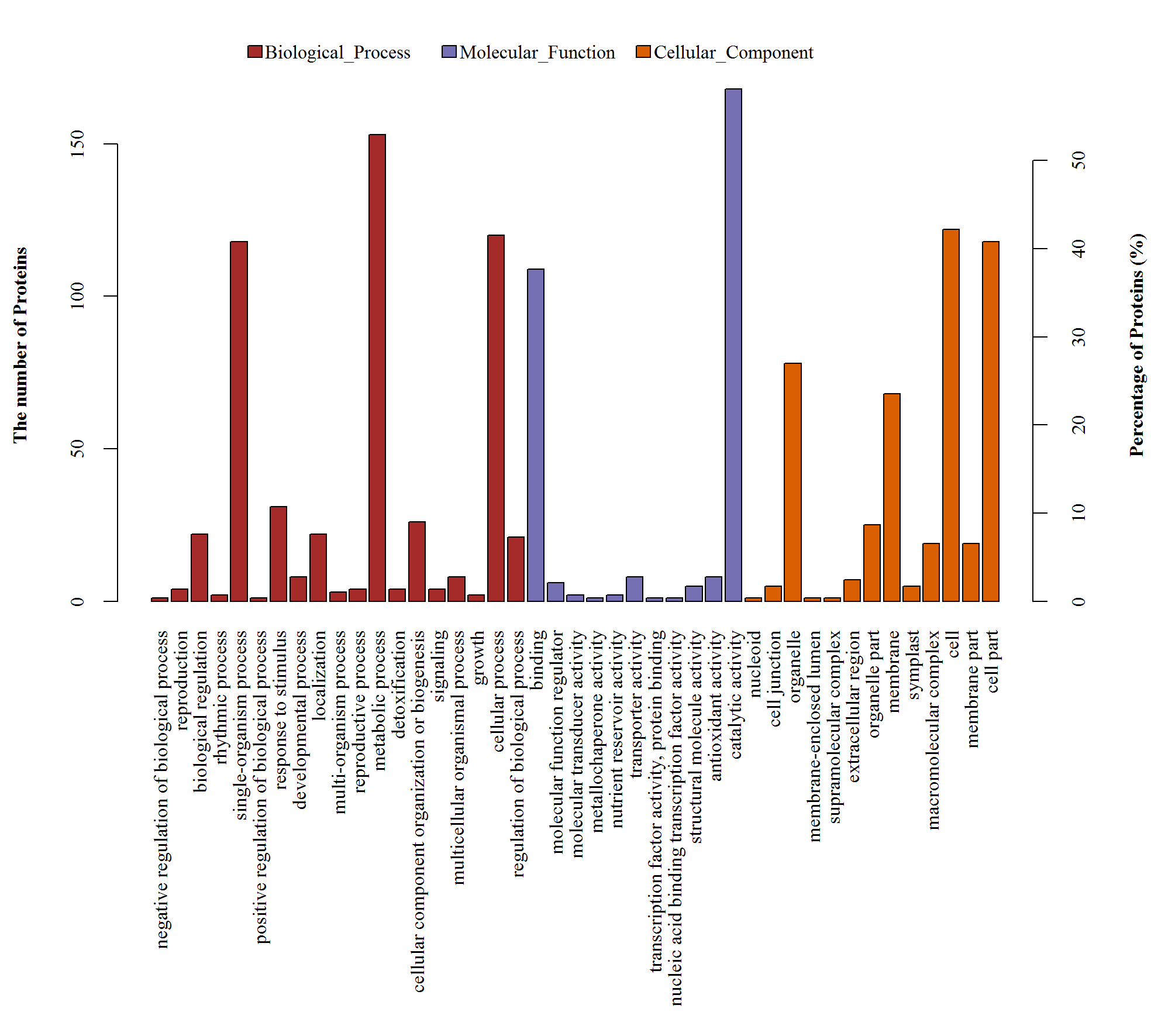
**

**A**

**
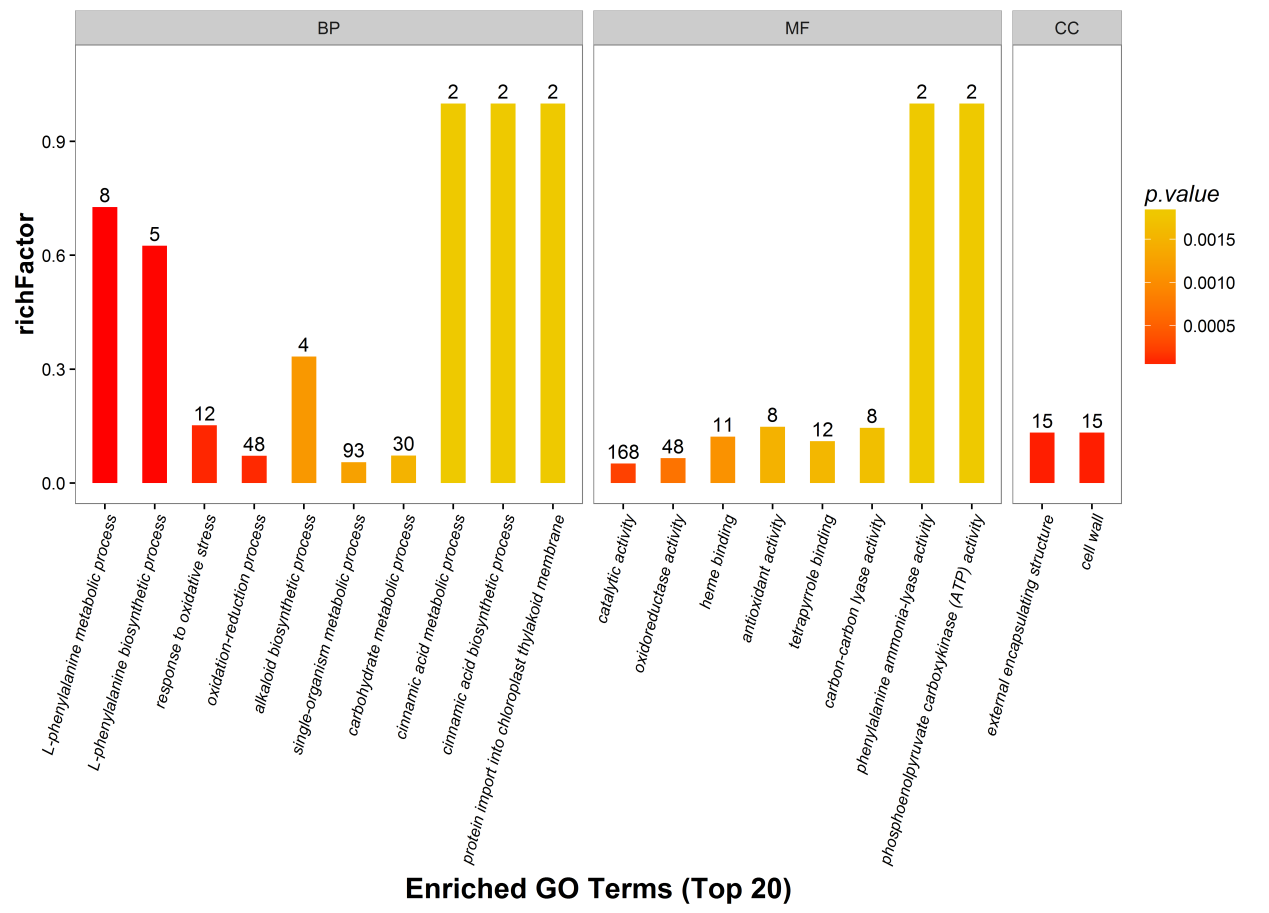
**

**B**


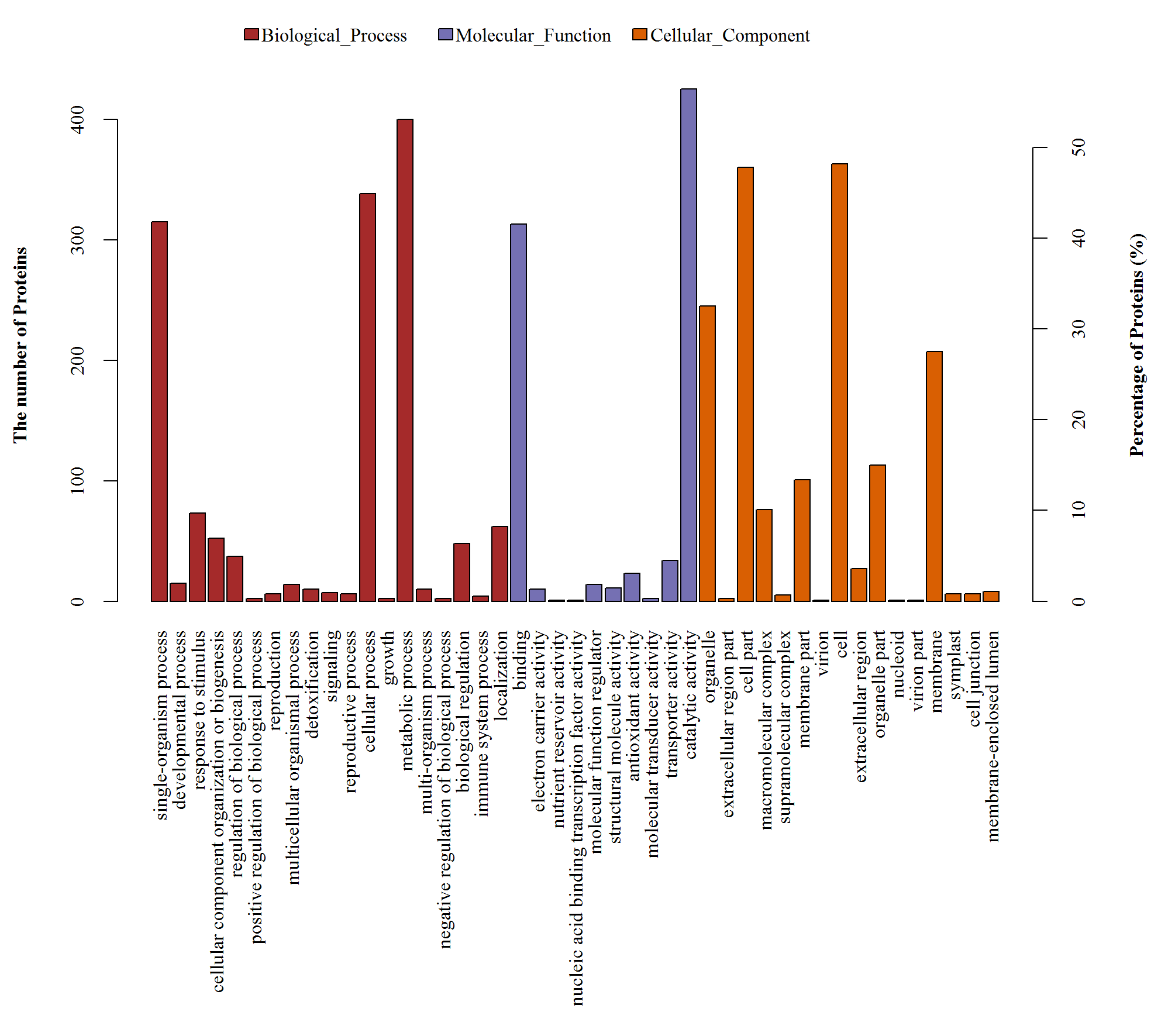


**C**

**
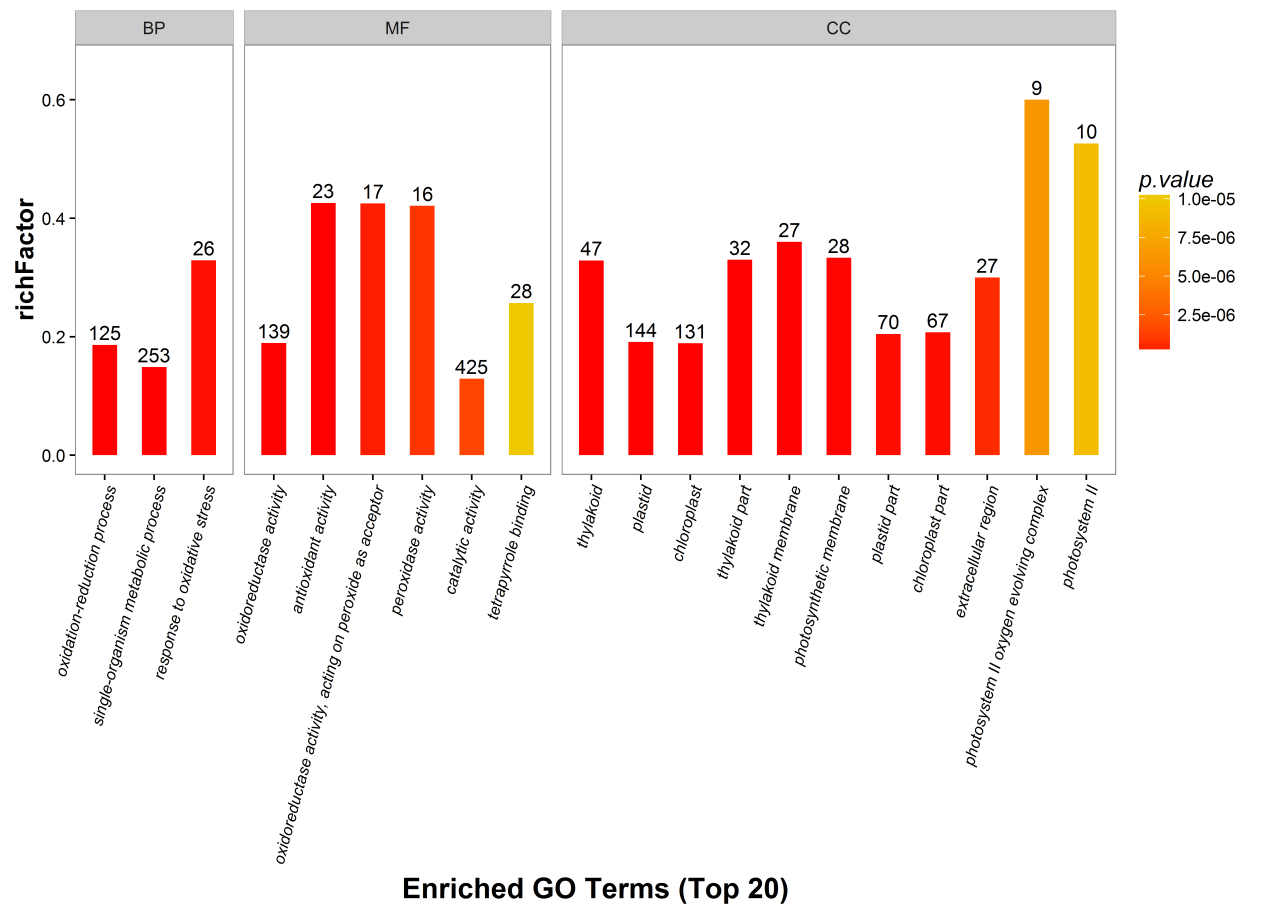
**

**D**
