# Supplementary figures and images for: Combination of iTRAQ proteomics and RNA-seq transcriptomics reveals multiple levels of regulation in phytoplasma-infected Ziziphus jujuba Mill
Source: Hortic Res. 2017 Dec 27;4:17080–. doi: 10.1038/hortres.2017.80 (PMC5744194; doi:10.1038/hortres.2017.80)

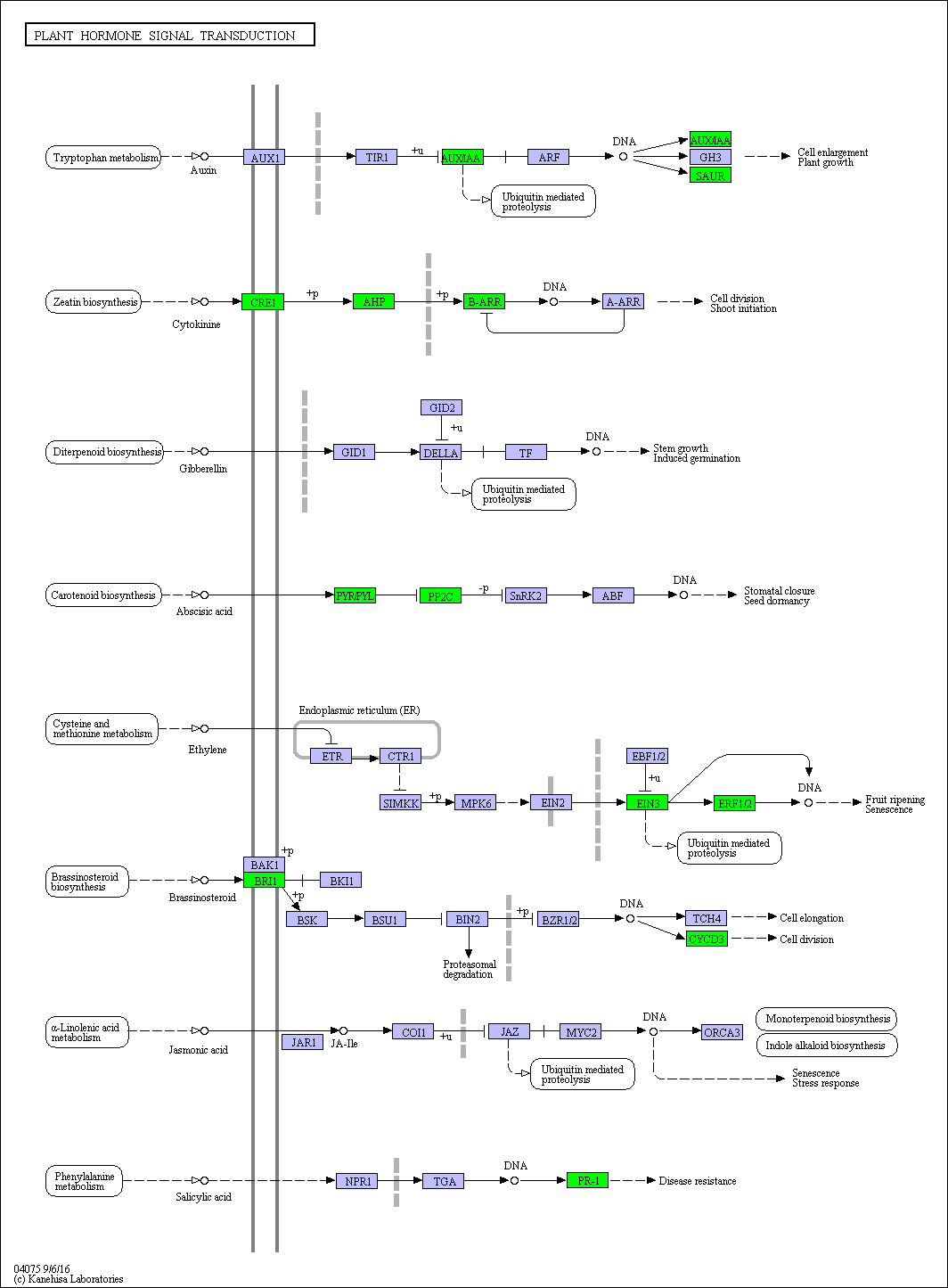

Supplement: Supplementary Figure S-3 [file hortres201780-s3.png]

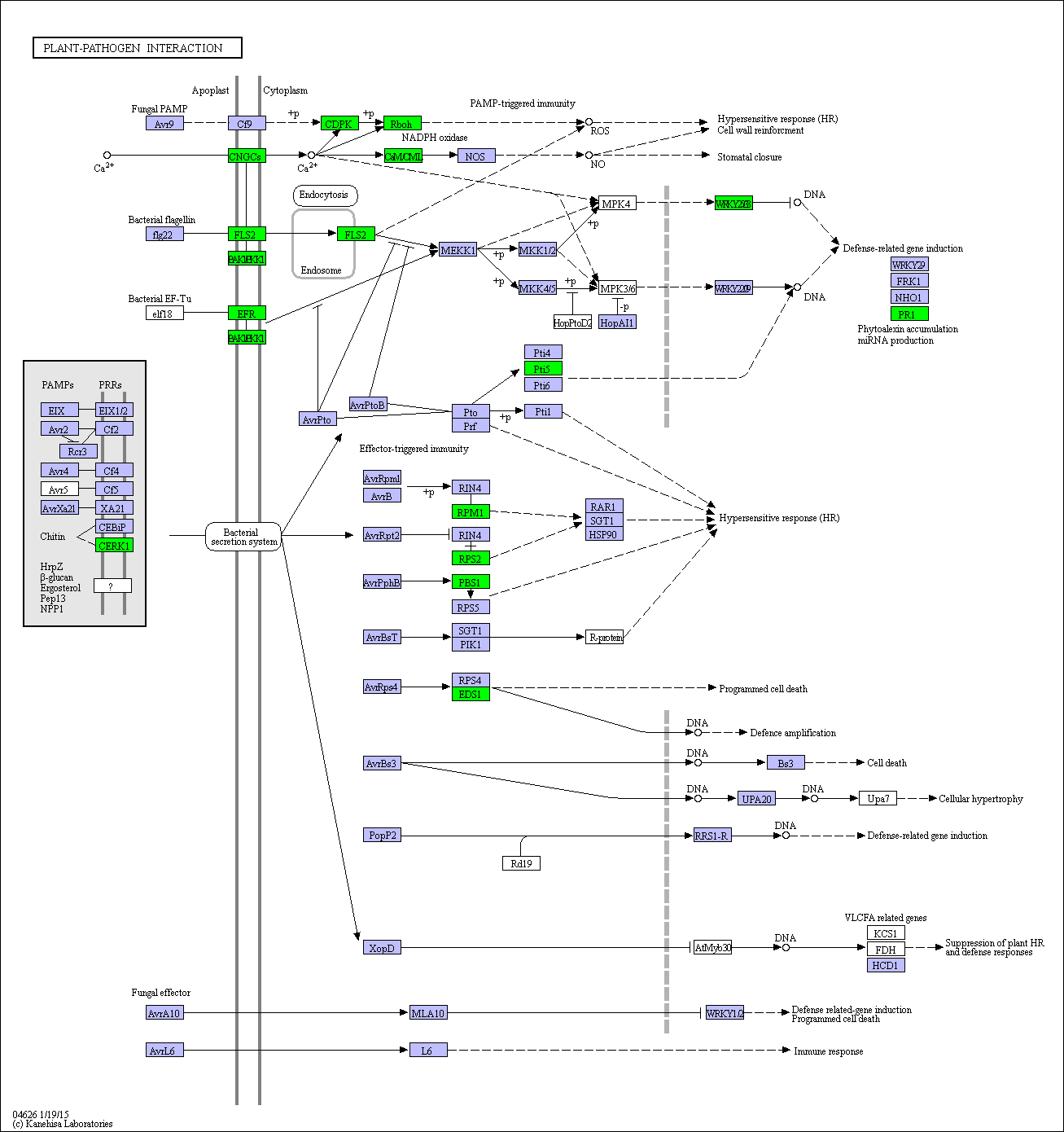

Supplement: Supplementary Figure S-4 [file hortres201780-s4.png]
