# Supplementary material for: Combination of iTRAQ proteomics and RNA-seq transcriptomics reveals multiple levels of regulation in phytoplasma-infected Ziziphus jujuba Mill
Source: Hortic Res. 2017 Dec 27;4:17080–. doi: 10.1038/hortres.2017.80 (PMC5744194; doi:10.1038/hortres.2017.80)
Supplement: Supplementary Table S-1 [file hortres201780-s5.docx]

**Table S-1. Primers for q-RT-PCR of DEGs at 37 WAG**

| Gene ID | Function description | Primer(5' to 3') |  |
| --- | --- | --- | --- |
| XLOC_000998 | auxin-responsive protein IAA | F: CTACAAAAGCCTCGCCAAAGC | |
|  |  | R: TGACCAGGAACAGCGTTTGA | |
| XLOC_010335 | auxin-responsive protein IAA | F: TCGGAAGGGACATGACAAAGA | |
|  |  | R: GCCACCCAACAAGTACAGCAT | |
| XLOC_004026 | auxin-responsive protein IAA | F: GATGGTGATTGGATGCTTGTTG | |
|  |  | R: CCTTTCATGATCCTAAGCCTTTTG | |
| XLOC_015148 | auxin-responsive protein IAA | F: GATGGTGATTGGATGCTGGTT | |
|  |  | R: CATAATTCGCACCCTCTTGCA | |
| XLOC_017103 | two-component response regulator ARR-B family | F: AGCCACTTCATCCTCCACTCA | |
|  |  | R: CCGAGGACTGGCTTGGTTAC | |
| XLOC_003747 | two-component response regulator ARR-B family | F: GGGTCGCCAAGTTTGATGAT | |
|  |  | R: CGCTTGAAGGCATCGATCTT | |
| XLOC_015928 | SAUR family protein | F: GTCAGCCTTCATTTCAGGAATTG | |
|  |  | R: TTGTGAGACCACCCATTGGAT | |
| XLOC_014375 | SAUR family protein | F: GGTTTTGGTGGGATTTGGAA  R: TCACCTCTCAAACTCTCCCTACAA | |
| XLOC_015929 | SAUR family protein | F: TTCCGCTCTTTAACATCAATGC  R: CTCTCCAAGCAAACACCACTCTT | |
| XLOC_014045 | SAUR family protein | F: TTGCCATGTTGAGGAGTTTAGGT | |
|  | Actin | R: TGGTGATGGAGGGACTTTTCC  F: CTTGCATCCCTCAGCACCTT  R: TCCTGTGGACAATGGATGGA | |

|  |
| --- |
|  |
|  |
